# Supplementary material for: Effects of Dezocine on the Reduction of Emergence Delirium after Laparoscopic Surgery: A Retrospective Propensity Score-Matched Cohort Study
Source: J Pers Med. 2023 Mar 28;13(4):590. doi: 10.3390/jpm13040590 (PMC10143985; doi:10.3390/jpm13040590)
Supplement: Supplementary file 1 [file jpm-13-00590-s001.zip › jpm-2253691-supplementary.pdf]

Table S1. RR of the Primary Outcome in the Propensity Score-Matched Cohort.

	Absolute Risk Difference, % (95% CI)	RR (95% CI)
**Emergency delirium**		
Dezocine group	−6.1 (−12 to −0.2)	0.63 (0.18 to 0.74)
Non-dezocine group	[Reference]	[Reference]
